# High‐resolution Doppler ultrasound in systemic sclerosis: Analysis of digital arteries and nailfold microvasculature using 18‐5 MHz and 33‐9 MHz probes

**DOI:** 10.1111/1756-185X.14422

**Published:** 2022-08-12

**Authors:** Riccardo Picasso, Pietro Bica, Federico Pistoia, Federico Zaottini, Sara Sanguinetti, Francesca Bovis, Marta Ponzano, Carmen Pizzorni, Sabrina Paolino, Alberto Sulli, Emanuele Gotelli, Carlo Martinoli, Maurizio Cutolo

**Affiliations:** ^1^ IRCCS Ospedale Policlinico San Martino Genoa Italy; ^2^ Laboratory of Experimental Rheumatology and Academic Division of Clinical Rheumatology, Department of Internal Medicine and Specialties (DIMI) University of Genoa Genoa Italy; ^3^ Department of Experimental Medicine (DIMES) University of Genoa Genoa Italy; ^4^ Department of Health Sciences (DISSAL) University of Genoa Genoa Italy

**Keywords:** connective tissue diseases, nailfold videocapillaroscopy, scleroderma patterns, systemic sclerosis, ultrasound

## Abstract

**Introduction:**

Newly developed Doppler techniques enable the sampling of slow vascular flows and the extrapolation of spectral parameters in distal arterioles. The aim of this study was to investigate the role of spectral analysis performed by means of ultra‐high frequency ultrasound (US) in the evaluation of the peripheral vascular bed of systemic sclerosis (SSc) patients.

**Methods:**

Both hands of 33 patients affected by diffuse cutaneous SSc and 34 volunteers were evaluated with a US machine equipped with 33‐9 MHz and 18‐5 MHz transducers. Proximal resistive index and the peak systolic velocity (pRI and pPSV, respectively), were calculated at the level of the second interdigital artery. The distal resistive index (dRI) was calculated at the level of a nailfold arteriole of the third finger. All SSc patients had been previously divided into 4 subgroups according to their nailfold videocapillaroscopic (NVC) patterns following accepted criteria.

**Results:**

SSc patients showed a significantly slower systolic velocity at the level of the second interdigital artery (pPSV [SD] = 8.38 [3] cm/s vs pPSV [SD] = 11.14 [4.5] cm/s; *P* = .005) and a higher dRI (dRI [SD] = 0.65 (0.14) vs dRI [SD] = 0.57 [0.11); *P* = .0115). No differences were found between the pRI values measured in the SSc patients and those of the controls (pRI [SD] = 0.76 [0.11] vs pRI [SD] = 0.73 [0.12]; *P* = .359]. The subgroup analysis did not show any significant difference when pPSV, pRI and dRI were compared among NVC morphological patterns.

**Conclusion:**

High‐resolution Doppler analysis of digital distal arterioles may disclose subtle abnormalities in the downstream microvasculature of SSc patients that could be missed when the examination is performed at a more proximal level and/or using lower Doppler frequencies.

## 1 INTRODUCTION

Systemic sclerosis (SSc) is a rare connective tissue disease characterized by vascular hyper‐reactivity and fibrotic changes affecting the skin and visceral organs. Most evidence suggests that microvascular injury plays a key role in SSc pathogenesis, and processes like vasospasm, impaired angiogenesis, increased platelet aggregation, and intimal fibrosis all represent hallmarks of the disease, even in its early phases.^1^, ^2^ Nailfold videocapillaroscopy (NVC) is an increasingly more used method for the evaluation of the peripheral vascular bed of rheumatological patients, and the identification of abnormal nailfold capillaries plays an acknowledged role in SSc diagnosis.^3^ In addition, specific NVC findings allow for the distinction between 3 different stages of the disease (ie early, active and late) and, in certain contexts, these findings may assist clinicians in targeting therapeutic decisions.^4^ However, NVC only enables a morphological evaluation of nailfold capillaries and the same pattern of alterations may be demonstrated in people with different rheumatological diseases or in healthy subjects.^5^ Doppler ultrasound (US) and spectral analysis of vessels are established methods for the clinical evaluation of several conditions determining fibrosis and parenchymal subversion of visceral organs, allowing for the extrapolation of several parameters that can provide essential information about disease progression and the response to therapy, such as arterial peak systolic velocity (PSV) and resistive index (RI).^6^, ^7^ PSV corresponds to each tall “peak” in the spectral Doppler waveform, and its value can vary depending on vessel properties (high‐ or low‐resistance) and pathological processes (eg vessel stenosis or occlusion); RI is the difference between the peak systolic and end‐diastolic flow velocities divided by the PSV and expresses the resistance and the vascular compliance in a pulsatile vascular system. Recent technological advancements in US equipment, with the introduction of ultra‐high frequency probes (frequency bands >30 MHz) and the progressive refinement of image‐processing algorithms, have opened up new perspectives for the evaluation of sub‐millimetric structures in very distal districts. In addition, newly developed Doppler techniques, by means of specific motion‐suppression algorithms that isolate and eliminate clutters, now enable the sampling of slow vascular flows and the extrapolation of spectral parameters in distal arterioles.^8^, ^9^ On this basis, the aim of our study was to investigate the potential role of spectral analysis performed by means of ultra‐high frequency US in the evaluation of the peripheral vascular bed of SSc patients.

## 2 MATERIALS AND METHODS

### 2.1 Investigated populations

Both hands of 33 patients with SSc (6 male, 27 female; mean age [SD] = 63.9 [14] years) and 34 healthy controls (8 male, 26 female, mean age [SD] = 48 [1.14] years) were evaluated using US by an experienced sonologist with specific skill in musculoskeletal and vascular imaging. The examiner was blinded to the clinical status of the subjects. All patients fulfilled the 2013 American College of Rheumatology / European Alliance of Associations for Rheumatology diagnostic criteria for SSc and had been previously divided into 4 subgroups (ie scleroderma‐like, early, active and late patterns) according to NVC findings as described in the literature.^10^, ^11^ NVC was performed by an experienced rheumatologist not more than 1 month before the US examination. Patients with active digital ulcers or ulnar artery occlusion were excluded from the study due to the impossibility to perform US on damaged skin. All the concomitant therapies were recorded and symptomatic compounds, influencing the peripheral blood flow (ie endovenous iloprost), were withdrawn 10 days before the US examination. This study was approved by the local ethics committee (CER Liguria: 5/2022—DB id 12 123) and informed consent was obtained from all patients.

### 2.2 US evaluation

**FIGURE 1:**
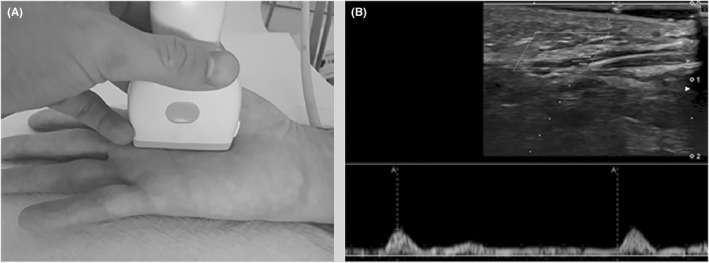
Peak velocity and proximal resistive index. (A) Photograph demonstrates the position of the probe at the third interdigital space. (B) Spectral analysis performed with an 18‐5 MHz ultrasound transducer

**FIGURE 2:**
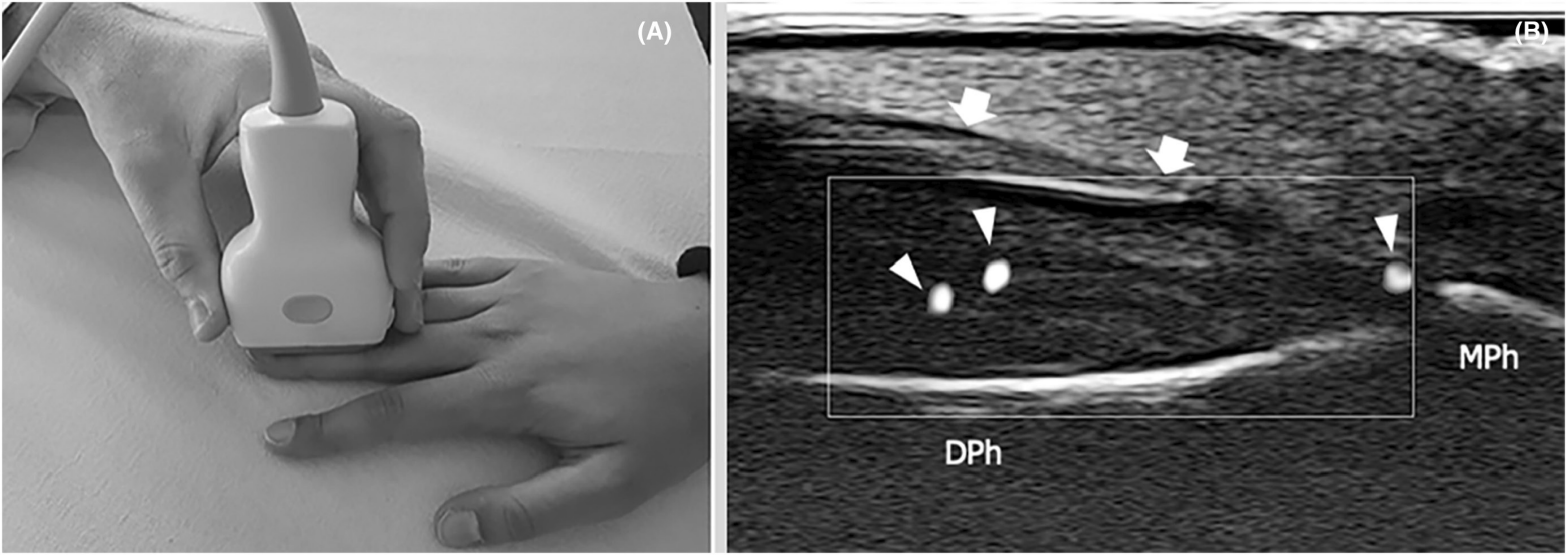
Distal resistive index. (A) Photograph demonstrates the position of the probe at the level of the dorsal aspect of the distal phalanx. (B) Long axis 33‐9 MHz ultrasound image demonstrates several small vascular spots (arrowheads) in the soft tissues underneath the nail (arrows). MPh, middle phalanx; DPh, distal phalanx

US was performed on both hands of the patients and controls, at the same hour of the day (between 14:00 and 18:00 hours) after a period of acclimatization in a room with a controlled temperature. Patients were invited to sit in front of the examination bed, with both hands laying on the bed. A US machine, equipped with 33‐9 MHz (frequency band for Doppler imaging, 10–18 MHz) and 18‐5 MHz (frequency band for Doppler imaging, 5–11 MHz) transducers (Aplio i800, Canon Medical System, Otawara, Japan), was used by the same sonologist for the evaluation of patients and controls. For both hands, the RI and the PSV were calculated at the level of the second interdigital artery (proximal resistive index and peak velocity, pRI and pPSV, respectively), orienting the 18‐5 MHz transducer along the long‐axis of the vessel on the palmar aspect of the third interdigital space. The interdigital artery was at first observed running approximately parallel to the skin and then changing its course, traveling obliquely from the surface to deeper down when approaching the base of the finger. Spectral analysis was performed at the level where the vessel presented the most oblique course and after having directed the US beam to obtain an incidence angle of 60° with the artery inferior (Figure 1); given its depth relative to the skin, the interdigital artery was not suitable for examination with the 33–9 Mhz transducer, so both the pRI and pPSV were calculated using the 18–5 Mhz transducer. Next, the distal RI (dRI) was calculated performing Doppler spectral analysis at the level of a nailfold arteriole of the third finger. In contrast to the interdigital artery, to study the nailfold and to calculate the dRI, we employed the 33–9 MhZ probe given its higher spatial resolution compared to the 18–5 transducer and its ability to show very small vessels with the use of a newly developed Doppler technique. The nailfold was initially investigated in a longitudinal plane, placing the 33‐9 MHz probe on the dorsal aspect of the middle and distal phalanxes of the third fingers. After the activation of microvascular flow settings, the color box was shifted to the soft tissue beneath the hyperechogenic profile of the nail and dRI was calculated in one of the small regional arterioles (Figure 2). At this level, the PSV was not measured due to the impossibility of obtaining the images of nailfold vessels in their long‐axis and adjusting the incidence angle between the US beam and the blood flow. On the other hand, the calculation of the dRI was possible as this is a purely adimensional parameter and its measurement does not require any rectification of the incidence angle. During the US examination, a large amount of gel was applied between the probe and the skin of the patients in order to avoid any alteration of the results due to soft tissue compression. In regard to the US parameters, the mean of the 2 measurements, respectively collected from the right and left hand, was considered for analysis. When it was not possible to measure a parameter in one of the patient's 2 hands, we took into account the measurement that had already been collected from the other hand as the mean value.

### 2.3 NVC examinations

NVC was performed using a 200× optical probe and images were detected and stored by means of ad‐hoc software (DS‐Medica, Milano, Italy). The examinations were performed in a room with a controlled temperature and a drop of immersion oil was applied to the nailfold to maximize the translucency of the keratin layer. All the patients' fingers, with the exception of the thumbs, were examined to detect the overall NVC pattern. Of note, the “scleroderma‐like” pattern is a transition pattern between the next SSc NCV patterns (mainly observed in mixed connective tissue diseases), the “early” NVC pattern is characterized by a relatively well‐preserved capillary arrangement and by numerous capillaries, whereas a moderate loss of capillaries and abnormal distribution in the nailfold typify the “active” NVC pattern. Finally, in the “late” NVC pattern, a severe loss of capillaries with extensive avascular areas and total disorganization of the normal capillary array are commonly observed.

### 2.4 Statistical analysis

Metric data are presented as mean ± standard deviation (SD) and range (minimum–maximum). To investigate differences among the NVC subgroups and between the patients and the controls, we applied analysis of variance or Kruskal‐Wallis test as appropriate, using the Kolmogorov–Smirnov test to verify normality. *P* values lower than .05 were considered statistically significant. Pearson's correlation coefficient was calculated between the US flow parameters of all the patients.

## 3 RESULTS

### 3.1 Demographic data and NVC examination

Demographic characteristics are summarized in Tables 1 and 2. All the patients had previous episodes of Raynaud's phenomenon and 82% of them had been treated with iloprost. The collection of the US parameters was successfully performed in most patients and in all the individuals of the control group. More specifically, it was possible to calculate dRI in 26 (78.8%) and the pRI and pPSV in 32 cases (97%) from the patients' group. The detection and sampling of the nailfold vessel flow was generally easier in the controls than in the patients, probably as a consequence of the disease‐related depletion of the distal vascular bed which occurs in people with SSc. The nailfold NVC categorized the patients as follows: *n* = 7, scleroderma‐like pattern; *n* = 4, early pattern; *n* = 14, active pattern; and *n* = 8, late pattern.

**TABLE 1:** Demographic characteristics of the investigated population

	Overall N = 67	Cases n = 33	Controls n = 34
Gender, n (%)			
Male	14 (21%)	6 (18%)	8 (24%)
Female	53 (79%)	27 (82%)	26 (76%)
Age, mean (SD)	55.90 (16.11)	63.97 (14.05)	48.06 (14.12)
BMI, mean (SD)	23.53 (3.75)	24.01 (4.62)	23.04 (2.60)
VCS pattern, n (%)			
Scleroderma‐like	‐‐‐	7 (21%)	‐‐‐
Early	‐‐‐	4 (12%)	‐‐‐
Active	‐‐‐	14 (42%)	‐‐‐
Late	‐‐‐	8 (24%)	‐‐‐
Raynaud phenomenon, n (%)	‐‐‐	33 (100%)	‐‐‐
Raynaud's duration, mean, y	‐‐‐	16	‐‐‐
Diabetes	‐‐‐	1 (3%)	1 (3%)
Arterial hypertension	‐‐‐	15 (45%)	8
CAD	‐‐‐	1 (3%)	‐‐‐
Smoking	‐‐‐	5 (15%)	6 (17%)

Abbreviations: BMI, body mass index; CAD, coronary artery disease; SD, standard deviation; VCS, videocapillaroscopy.

**TABLE 2:** Antibody profiles and therapy of the investigated population

	Overall N = 67	Cases n = 33	Controls n = 34
ACA	‐‐‐	13 (39%)	‐‐‐
Anti‐Scl‐70 Ab	‐‐‐	12 (36%)	‐‐‐
APA	‐‐‐	4 (12%)	‐‐‐
Anti‐PM/Scl100 Ab	‐‐‐	1 (3%)	‐‐‐
AMA	‐‐‐	2 (6%)	‐‐‐
Anti‐Ro52 Ab	‐‐‐	1 (3%)	‐‐‐
Anti‐CCP Ab	‐‐‐	1 (3%)	‐‐‐
RF	‐‐‐	2 (6%)	‐‐‐
Anti‐NOR90 Ab	‐‐‐	1 (3%)	‐‐‐
ACPA	‐‐‐	1 (3%)	‐‐‐
Antiphospholipid Ab	‐‐‐	1 (3%)	‐‐‐
Anti‐Ku Ab	‐‐‐	2 (6%)	‐‐‐
Anti‐PM/Scl‐75	‐‐‐	1 (3%)	‐‐‐
ASA = 1, n (%)	‐‐‐	28 (85%)	‐‐‐
Aminaphtone, n (%)	‐‐‐	27 (82%)	‐‐‐
Methotrexate, n (%)	‐‐‐	7 (21%)	‐‐‐
Prednisolone, n (%)	‐‐‐	3 (9%)	‐‐‐
Hydroxy hydroquinone, n (%)	‐‐‐	2 (6%)	‐‐‐
Cyclosporine, n (%)	‐‐‐	6 (18%)	‐‐‐
Mycophenolate mofetil, n (%)	‐‐‐	8 (24%)	‐‐‐
VitD, n (%)	‐‐‐	32 (97%)	‐‐‐
ACE inhibitor, n (%)	‐‐‐	18 (55%)	‐‐‐
Ca antagonist, n (%)	‐‐‐	2 (6%)	‐‐‐
PPI, n (%)	‐‐‐	25 (76%)	‐‐‐
Endothelin receptors antagonist, n (%)	‐‐‐	9 (27%)	‐‐‐
Iloprost, n (%)	‐‐‐	27 (82%)	‐‐‐
Nintedanib, n (%)	‐‐‐	2 (6%)	‐‐‐

Abbtreviations: ACA, anticentromere antibodies; Ab, antibodies; APA, antipolymer antibodies; PM: polymyositis; AMA, antimitochondrial antibodies; CCP, cyclic citrullinated peptide; RF, rheumatoid factor; NOR, nucleolus organizer region; ACPA, anti‐citrullinated protein/peptide antibodies; ASA, acetylsalicylic acid; VitD, vitamin D; ACE, angiotensin‐converting enzyme; Ca, calcium; PPI, proton pump inhibitors.

### 3.2 US evaluation

Tables 3 and 4 summarize the results of the study. The US examination with the spectral analysis took, on average, 20 minutes per patient. SSc patients demonstrated a significantly slower systolic velocity at the level of the second interdigital artery (pPSV [SD] = 8.38 [3] cm/s vs pPSV [SD] = 11.14 (4.5) cm/s; *P* = .005) and a higher dRI (dRI [SD] = 0.65 [0.14] vs dRI [SD] = 0.57 [0.11]; *P* = .0115) compared to controls. No differences were found between the pRI values measured in the patients and those of the controls (pRI [SD] = 0.76 [0.11] vs pRI [SD] = 0.73 [0.12]; *P* = .359). The subgroup analysis did not show any significant difference when pPSV, pRI and dRI were compared among NVC categories. Pearson's test demonstrated no significant correlation between the patients' flow indices.

**TABLE 3:** Mean (SD) of US parameters among cases/controls and by NVC groups; *P* values for US parameters differences among NVC groups and between cases and controls. Cases showed higher resistive index (*P* = .0115) and slower systolic velocity at the level of the second interdigital artery (*P* = .005)

	Cases N = 33	VCS = 0 n = 7 (21%)	VCS = 1 n = 4 (12%)	VCS = 2 n = 14 (42%)	VCS = 3 n = 8 (24%)	Controls n = 34	VCS classes	Cases vs controls
	Mean (SD)	Mean (SD)	Mean (SD)	Mean (SD)	Mean (SD)	Mean (SD)	*P* value	*P* value
dRI	0.65 (0.14) n = 26	0.67 (0.11) n = 6	0.83 (0.22) n = 2	0.64 (0.14) n = 13	0.59 (0.14) n = 5	0.57 (0.11) n = 33	.2540	.0115
pRI	0.76 (0.11) n = 32	0.73 (0.11) n = 7	0.72 (0.07) n = 4	0.76 (0.09) n = 14	0.81 (0.14) n = 7	0.73 (0.12) n = 34	.5354	.3596
PV, cm/s	8.38 (3.00) n = 32	7.30 (2.57) n = 7	8.51 (4.03) n = 4	9.00 (3.36) n = 14	8.16 (2.20) n = 7	11.14 (4.50) n = 34	.6868	.0050

Abbreviations: VCS, videocapillaroscopy; SD, standard deviation; dRI, distal resistive index; pRI, proximal resistive index; PV, peak velocity.

**TABLE 4:** Pearson's test demonstrated no significant correlation between the patients' flow indices.

Pearson's correlation coefficient (R)			
Significance (*P*)			
Number of observations (n)			
	Proximal resistive index	Peak sistolic velocity	Distal resistive index
Proximal resistive index	1.00000 (R) 32 (n)	0.19500 (R) .2848 (*P*) 32 (n)	0.15274 (R) .4563 (*P*) 26 (n)
Peak sistolic velocity	−0.19500 (R) .2848 (*P*) 32 (n)	1.00000 (R) 32 (n)	−0.07717 (R) .7079 (*P*) 26 (n)
Distal resistive index	0.15274 (R) .4563 (*P*) 26 (n)	−0.07717 (R) .7079 (*P*) 26 (n)	1.00000 (R) 26 (n)

## 4 DISCUSSION

Although SSc is considered an idiopathic, small‐vessel vasculopathy, the production of auto‐antibodies and fibroblast dysfunction are all thought to represent the main pathogenic processes leading to clinical manifestations. In particular, it has been speculated that the primum movens of SSc consists of an auto‐immune reaction directed against the small vessels and characterized by the production of anti‐endothelial auto‐antibodies (which may be demonstrated in the sera of up to 84% of patients with SSc) and the activation of T‐cells releasing proteolytic granzymes.^12^, ^13^ This leads to a widespread obliterative vasculopathy with the predominant involvement of distal arterioles and capillaries, eventually resulting in diffuse ischemic damage, involving both the skin and visceral organs.

The relevance of peripheral vasculopathy in SSc pathogenesis has led to a growing interest in developing a tool enabling an in‐depth evaluation of the distal vascular district of patients affected by this condition. Recently, the advent of high‐resolution US and the refinement of Doppler technology have opened promising perspectives about the possible extrapolation of quantitative parameters reflecting the status of distal arteries in SSc patients.^14^ Our work is based on the assumption that the increased sensitivity of Doppler imaging could provide information about the status of the microvasculature at the most peripheral level of extremities, and that disease‐related changes affecting the distal bed could be better characterized when the Doppler signal is sampled closer to the area of expected abnormalities. On the other hand, the detection of slow blood flows in nailfold capillaries requires ultra‐high frequencies and specific motion‐suppression algorithms to discriminate very slow, almost stationary, flows from interstitial fluid movements.

To the best of our knowledge, this is the first study showing the disease‐related variations of nailfold blood flow detected by means of US spectral analysis in SSc patients. In our patient group, US showed an increased dRI at the level of nailfold arterioles. Interestingly, the slowing‐down of PSV was the only alteration in the blood flow dynamics detected at the level of the interdigital artery, whereas pRI was unaffected. These results may be partially explained by consideration of the fact that vasculopathy in SSc has been demonstrated to involve predominantly the most peripheral downstream circulation, more specifically arterioles and capillaries,^15^, ^16^, ^17^ and that the RI reflects the resistances to blood flow determined by the compliance of vessels located distal to the site of measurement. In other terms, the more proximal the sampling site, the more unselective and undiscriminating the analysis of blood flow since measurements derive from non‐specific analysis of affected (minor) and nonaffected (prevalent) microvascular areas. In this perspective, the calculation of pRI at the level of the interdigital artery may not allow for the detection of a pathological process affecting the small arterioles of the nailfold, as the augmented resistances in the distal district could be partially mitigated by the normal compliances found in more proximal and unaffected vessels. These findings are in accordance with a recently published paper in which the authors were unable to demonstrate any correlation between the RI of digital arteries at the level of the proximal phalanx and nailfold perfusion.^18^ On the other hand, it is more difficult to explain the significant slowing‐down in the interdigital artery systolic velocity that was observed in patients. It could be hypothesized that the progressive depletion of the distal vascular bed may induce complex changes in the proximal district in an attempt to maintain perfusion, among which vasodilatation may be responsible for the regional decrease in blood flow velocity, but further investigations are necessary to confirm our findings.

However, our study has several limitations. First, we were able to recruit a small number of patients and it was not possible to extrapolate flow parameters from all of them. Second, no correlation between pRI, dRI and PSV and NVC categories was found, possibly due to the small number of samples considered in our analysis. In addition, we investigated only a small part of the parameters provided by spectral analysis, excluding other data, such as the diastolic velocity, which could have been able to provide additional clues to understanding the complex changes occurring in vessels of patients with SSc. Finally, we cannot exclude the possibility of the influence of some patients' therapies on flow parameters, as it was not possible to evaluate patients when they were not in their specific treatment cycle.

In conclusion, the ability to perform spectral Doppler analysis of small distal arterioles by means of ultra‐high frequency US transducers is opening new perspectives to disclose subtle abnormalities in the downstream microvasculature of SSc patients that could be missed when Doppler examination is performed at a more proximal level and/or using lower Doppler frequencies. Further studies are needed in order to investigate the ultimate role of the microvascular Doppler US in the assessment of peripheral vasculopathy in SSc patients and its potential in providing useful data in clinical diagnosis and follow up.

## CONFLICT OF INTEREST

The authors have no conflict of interest related to this work.
